# Editorial

**Published:** 1861-10

**Authors:** 


### Editorial.

MEDICAL SOCIETIES.—The local medical societies existing in most cities and large towns, which are accustomed to hold meetings once a week or once a month, have ever found it difficult to keep up a full or even a moderate attendance during the summer months. The increased amount of business at that season of the year, and the short evenings, are obstacles in the way of the punctual attendance of members. During the past summer, however, the excitement consequent upon the unhappy condition of the country, has furnished an additional obstacle in the way of maintaining social organizations.

For a few years past Chicago has had two medical societies, each in a tolerably active and prosperous state. Each has held its meetings once a month, and many members of the profession in the city belong to both. For the last three or four months it has been difficult to get a quorum together in either society, and in the Academy serious thoughts were entertained of disbanding altogether. But at the regular meeting held on the first Friday evening of this month, a new start was taken, and all needful preparations were made for resuming the business of the Academy with renewed energy and interest. Preparatory steps were taken to lop off all the dead branches, to revise the finances, and to enter upon medical and surgical investigations and discussions more systematically than before. A standing committee was appointed to present, in the form of a brief report, at each meeting, whatever there was new and important to be gleaned from our current medical literature. The *treatment of delirium tremens* was chosen as the subject for discussion, and a member was appointed to deliver the Annual Oration at the next meeting.

The Chicago Medical Society, which is much older than the Academy, has had but a thin attendance at its meetings for a few months past, though its members have at no time thought of giving up its organization. Doubtless it, too, will now exhibit new life; aud we would suggest that its meetings, from this time until spring, be held once a week, instead of once a month. There is no real jealousy between these organizations, and we trust they will both be active and prosperous at the time for the meeting of the American Medical Association, on the first Monday in June next.

---

WHAT AILS THEM?—The editors of the *Chicago Medical Journal*, who are also prominent members of the Faculty of Rush Medical College, seem to have been in a very unpleasant state of mind for many months past. Very few things appear to suit them. In one paragraph they give the government a slant about the war; in another they cast insinuations on the Army Board of Medical Examiners; in another they hint that some regimental surgeons have salivated their patients; and again it is blindly intimated that somebody has been appointed Surgeon to a regiment who had, at some time in his life, mistaken an inguinal hernia for a bubo. They are equally disconsolate and troubled about the manner in which the Army Board of Examiners for this State perform their duties, and in which we edit the EXAMINER, or even conduct our professional practice. We are sorry that our neighbors are in such a perturbed and unhappy state of mind, and the question has often been asked, "What ails them?" Without claiming any superior skill, we think the answer can easily be guessed out. Less than three years since three members of the Faculty of Rush Medical College resigned their places in that institution. Those three, included the men who for years had given nearly all the preliminary lectures before the commencement of each annual session, had performed all the labor of finding accommodations for students as they came into the city, and had published their medical journal, furnished most of the members of the faculty with copies gratuitously, and footed the bills out of their own pockets. Since these resignations, a large part of the labors just enumerated have fallen upon the present editors of the *Chicago Medical Journal*, and it is a fact well known to careful observers, that *much labor* and *little pay* always produces a very unpleasant *constitutional irritation* in certain temperaments. This, together with the fact that neither President LINCOLN nor GOV. YATES saw fit to place the medical department of the army, either in the nation or the State, directly under the supervision of the senior editor of that journal, is amply sufficient to explain their unhappy condition. We hope they will learn to bear their infirmities with more patience.

MAL-PRACTICE SUIT.—A decision in the case of FARRELL *vs.* CADWELL, in the Superior Court, before Judge MCALISTER, has just been rendered against the defendant, fixing the *ad damnum* at *ten thousand dollars*. The defendant was Dr. F. A. CADWELL, a somewhat notorious oculist and aurist of this city, and the plaintiff, a domestic in one of the hotels. The plaintiff averred in her declaration, that on the 13th of July, 1860, she applied to the defendant to remove an imperfection from her left eye, being a scar caused by small-pox; that the defendant pretended he could restore the eye to its original appearance; that the operation would not be painful, and would not keep plaintiff from her work longer than six or seven days; that it would not impair the right eye, which was then well and healthy. That in consequence of these representations, she submitted to the operation, previously paying the sum of $30, which he demanded in advance; that he conducted himself in an unskillful, ignorant and negligent manner; that he improperly cut and punctured the left eye, so that inflammation set in. That he afterwards did not visit the patient nor attend upon her; but carelessly and improperly caused her to be led out to his office, when she was in a weak and feeble state of health, whereby she took cold in her eyes, producing inflammation, whereby the left eye was lost; and afterward that he introduced into the right eye certain inflammatory drops, whereby the right eye was greatly injured, and its sight destroyed.

The defendant, on his part, alleged the disease was *staphyloma*, that he did not undertake to restore the eye to its original condition, but to reduce its size, in order to insert a glass eye over it; that he practiced ordinary, if not more than ordinary skill, for this purpose, and that the loss of the plaintiff's eye was the result of her own neglect, instead of his ignorance, neglect or mal-practice. The jury, after being out some sixteen hours, handed in their verdict, as above, when counsel for defense moved, in arrest of judgment, for a new trial.

Demonstrator of Anatomy.—In the September number of the Examiner we stated that the place of Dr. H. Wardner, formerly Demonstrator of Anatomy in the Medical Department of Lind University, but now Surgeon of the 12th Regiment of Illinois Volunteers, would be supplied during the present college term by Dr. J. S. Jewell. At the time, we supposed that such an arrangement had been completed, but the announcement was premature.

The place has since been filled by the appointment of Ernst Schmidt, M. D., of this city, but recently Professor of Pathology in the Humboldt Institute, of St. Louis. Prof. Schmidt is an accomplished scholar, and an experienced teacher, and we are quite sure that he will discharge the duties of his new position in such a manner as to confer honor, both upon himself and the University.

Chicago Sanitary Commission.—A citizen's meeting was held, in this city, on the 17th inst., at which the following gentlemen were appointed a Sanitary Commission: Hon. MARK SKINNER, President; H. E. SEELYE, Esq., Permanent Secretary; E. W. BLATCHFORD, Esq., Corresponding Secretary; Col. J. W. FOSTER, JAMES WARD, Esq., RALPH N. ISHAM, M. D., REV. Dr. TIFFANY, and Rev. Mr. PATTON.

We are not advised as to their sphere of duties, but presume they will co-operate with the National Sanitary Commission, and, directly, have in charge the welfare and sanitary care of our Illinois troops. And *ápropos* to which, we may mention that twenty additional nurses and two Sisters in charge, have been dispatched to St. Louis and Jefferson City, by the Sisters of Mercy of this city, making in all thirty-two able, intelligent, trained nurses sent hence by this Order to care for our sick and wounded in Missouri.

OPENING OF THE MEDICAL COLLEGES IN CHICAGO.—The Medical Department of Lind University opened its regular winter term on Monday evening, the 14th inst., by an introductory lecture from Prof. E. ANDREWS.

The Rush Medical College commenced its regular term, we believe, on Wednesday evening, the 16th inst.

We are informed that the prospects are good for fair classes in both institutions. The Faculties of both will do their best to give every student who comes to Chicago as good facilities for education as he can find any where else.

ACTION OF OPIUM ON THE GENITO-URINARY ORGANS.—Dr. GEO. B. WILLSON, of Pt. Huron, Mich., commenting* on Dr. WOODWARD's article on the above subject, originally published in the EXAMINER, remarks that he has frequently seen the muscular coat of the bladder so paralyzed by the dose of one-third of a grain of acetate of morphia as to necessitate the use of the catheter, and proposes its relief or avoidance by the coincident use of sweet spirit of nitre. The Dr. goes on to define his views of the therapeutic action of the drug, differing from Dr. WOODWARD, as to its true impression upon the kidney,—the latter, it will be remembered, attributing the "increased secretion from the kidneys to its relaxing properties," while Dr.

* In the *Boston Medical and Surgical Journal*, Oct. 17th, p. 209, Vol. LXV.

WILLSON claims that "it acts upon the kidneys, the skin, and every other part by which it is excreted, as a direct irritant." This irritation, increasing the secretion of water simply, also produces "the spastic contraction of the sphincter of the neck of the bladder"—before alluded to as paralysis of the muscular coats, and this, he remedies by the relaxant and anti-spasmodic action of the sweet spirit of nitre.

Dr. WILLSON concurs with Dr. WOODWARD as to the anaphrodisiac effects of opium, and adds, that its use persevered in, repeatedly, for several days or weeks at a time, and with intervals of the same, will produce atonic spermatorrhœa, during the intervals.

We hope the Dr. will favor our readers with a paper on the effects of opium on the procreative functions, agreeing with him, that "it is a shame and disgrace to the profession, that so important an article should be so little understood."

---

BOOKS AND PAMPHLETS RECEIVED.—In addition to the volumes, noticed in the proceeding pages, we also have to acknowledge the receipt of *The Pathology and Treatment of Venereal Diseases; Including the Results of Recent Investigations on the Subject*, a handsome octavo volume of pp.686, by the well-known lecturer on this subject in the New York College of Physicians and Surgeons, and translator of Ricord's Hunter, FREEMAN J. BUMSTEAD, M. D.

Also, the fifth American, from the seventh and revised London edition of TAYLOR'S *Medical Jurisprudence.* Also, *The Transactions of the Medical Society of the State of New York, for the year*, 1861.

The *London Lancet*, HAY'S *American Journal*, and the usual monthlies and weeklies are, also, received.

---

DR. GIBSON ADVISES for stomatitis materna as "nearly specific as quinine for intermittent fever," chlorate of potash, half drachm doses daily, and application of solution of tannate of zinc to the part affected. The use of chlorate of potash in this and all kinds of stomatitis is indeed "nearly a specific," and has long been so regarded and employed in this city.—*Amer. Med. Monthly.*

United States Sanitary Commission.—We have not as yet, received the volume of the *Transactions* of this body; but reprint the following extracts from the "Notes of a Preliminary Survey of the United States Force in the Ohio and Mississippi Vallies, by H. W. Bellow, D.D., President of the Commission," from one of our exchanges: *

"Though low, it (Cairo) is now neither damp, muddy, nor unhealthy. The water which stands in the plain a few inches deep, after a heavy rain, very soon, owing to the sandy character of the soil, disappears. Engines are at work, also, to drain the surplus water off into the river. The army has cleared away some thousands of stumps from the central plain of Cairo, and created a very fine parade of two or three miles long, and a mile or so broad. Col. Paine's regiment was chiefly active in this good work, which will prove of lasting service to Cairo. The general health of the place is testified to by an intelligent resident physician (a Virginian) as being better than at most points on the Ohio and Mississippi. Fever and ague does not abound, and there seemed to be a general testimony among the army surgeons there, that the health of the troops was as good as at any other point, where so many men are collected. The sick list showed us about 250 on their backs in a force of 6000, which, at the close of June and first of July, is not an excessive number. The open, airy character of Cairo, situated between two rivers, which act by their unequal currents as perpetual ventilators, save it from the influence of the malarious airs, which seem to blow over it, and produce their mischievous effects in the high lands beyond, on bluffs crowded with wood, as at Villa Ridge, clothed with a forest obstructing the free passage of winds, and occasioning, perhaps, by a cooler atmosphere, a precipitation of the poison at a particular level. Cairo proves more healthful than would be supposed from its apparently exposed position."

Dr. Bellows finds in too many instances, much inattention to cleanliness, carelessness on the part of the men, overcrowding, etc., which seem to be the almost universal faults among our volunteers at first. We cannot help quoting one case of gross want of judgment in locating a camp, and of apparently culpable remissness on the part of the medical officers. Speaking of the 22d Illinois regiment, in camp at Caseyville, he says:

"The 22d regiment, Col. Dougherty, is in a wretched condition. It is encamped only a half mile to the east of the 13th.

---

*The *Boston Med. and Surg. Journal*, October 17, 1861.

But it is in a valley, beneath very shady trees, and under the lee of some hills, all which combine to make the miasmatic atmosphere stagnate at the spot. as the winds have no circulation. They have been there only thirteen days, but have 250 men, out of about 900, more or less sick with camp dysentery. This is due in part to the situation, but in part also to the water, which is black and disgusting. It is taken from some pits sunk in a kind of a half stagnant gutter, in the other end of which the pigs are rooting. All the water they have is from this wretched source, and they have not enough even of this. Of course they mix worse rum with this bad water, and the men are poisoned.

"The hospital is in a room hired for the occasion, which is a perfect pig-sty for nastiness. The accommodations are only for, say five and twenty, and the sick are 250. The steward (for both surgeon and assistant were absent) had made fifty prescriptions to-day, and was not through yet. The camp has no hospital tent or stores. except what it borrows from the 13th. The surgeon of that regiment is also absent. There is evidently a gross neglect in these easy absences, granted at a time when no excuse should suffice to absent the doctor, who is so sadly wanted."

Speaking of the effect of the water upon the health of a large camp, Dr. Bellows says:

"It is evident that change of water, and especially *bad* water, is the most immediate and serious cause of illness in all western camps at this time. Pains enough are not taken to place the camps with reference to the vicinity of good water. The best water in Illinois was said to be found at a ridge running down from four miles below Alton, near the Junction, where broad and excellent camping and parade grounds existed. The colonels had *prospected* this place, and approved it; but were nevertheless—so I heard from a reputable source—ordered to remain where they were, and where they had actually suffered at first for want of *enough* water, because the contractors found the immediate neighborhood of Alton a more profitable place to meet their engagements in!"

Referring to the matter of discipline, the Commissioner says:

"Col. Turner said the great difficulty was in getting the men to obey officers no better than themselves, and often not as good. The officers might *persuade*, but did not know how to *command* men they associated with at home as equals. And this is the chief misfortune about the volunteers, and really raises the question, whether the men of one district would not be better officered from another. The colonel complained that it was very difficult to have the camp police, in respect to the use of sinks, carried out, and this was evident to several senses."

This is an evil which our Virginia experience must have done much to correct by this time; actual service in the field soon shows men the importance of obedience to their officers.

As a specimen of the operation of the red tape, we give the following:

"It is evident that the medical directors are in general too few, too old, or too inactive; that they do not go about and inquire into the wants of the surgeons and hospitals, and facilitate their accommodation with stores. The regiments at Caseyville, Cairo, Alton, had been visited by Dr. Taggart, who referred them to Dr. ——, who was with General McClellan. But all this roundabout inquiry compelled these urgent hospital wants to be referred to Springfield—a distant place—where orders were made out to be filled at Cincinnati, while at the time a medical director and purveyor both existed at St. Louis, with abundant stores, whence, at a distance of nine miles from Caseyville, twenty from Alton, and six hours or so from Cairo, all these wants could be in twenty-four hours fully met. I endeavored to bring this about; but the medical director at St. Louis is old and inactive, and past real usefulness; while Dr. Bailey, medical purveyor, no longer young, lives at Jefferson Barracks, where he is surgeon, and does the duties of this St. Louis post as extra service, which is all wrong. Young, active and efficient men are solely wanted in this important department. The lack of a regular inspector, U. S. A., flying through the camps, communicating information, and spurring on and facilitating official service, is most obvious."

Medicine and Surgery in the Army.—The U. S. Sanitary Commission have just published a circular, drawn up by Prof. Van Buren of New York, recommending the use of quinine in our armies, as a prophylactic against the malarious fevers of the regions they are now, or will be operating in during the war. The following extracts we re-print from the *Amer. Med. Times:*

"Dr. Van Buren alludes to his own experience while on the medical staff of the U. S. Army in Florida. A serious outbreak of miasmatic disease occurred at the station, and the stock of quinine being exhausted, a substitute was prepared with whisky, the bark of the common dogwood, wild cherry bark, and a small quantity of quinine, which reduced the number of relapses, and mitigated the attacks.

The British naval authorities have long been impressed with these facts, and have acted accordingly. Dr. Bryson states in the Navy Medical Reports, (No. xv.) that—

"It has long been a standing rule in the Navy, enjoined by the 9th Article of the Surgeon's Instructions, that when men are to be sent on shore in tropical climates, to procure wood and water, or on other laborious duties, the surgeon, if he consider it advisable, is to recommend for each man, previously to his leaving the ship, in the morning, a drachm of powdered bark (Peruvian,) in half a gill of wine, and the like quantity of wine after the mixture; or, if there be no wine on board, one-eighth of a gill of spirits, mixed with the fourth of a gill of water, is to be used in lieu of it; and the same proportion of each is to be given to the men on their return to the ship in the evening."

The British Army has been similarly provided, and the medical officers directed to employ quinine as a prophylactic. During the war of the Crimea, the Medical Director of the Army wrote as follows to the Inspector General of Hospitals:

"With reference to previous letters on the subject of administering quinine, and other preparations of bark, as prophylactic remedies, I have the honor again to draw your attention to the matter. From all I have learnt I am persuaded that the number of cases of fever would be diminished by such a course. So convinced am I, especially by the results of the experience of naval medical officers, of the benefits arising from the prevention plan, when followed in localities in which remittent and intermittent fevers are likely to prevail, that I have taken care to provide ample supplies of quinine in anticipation of every possible demand for that article. Having now at command sufficient of this drug, specially provided for that service, to furnish five grains per diem to every member of a force of 35,000 men, I beg you will take such measures as you think proper, with a view to induce the medical officers to employ that remedy, in the hope that it may prove useful in warding off attacks of fever, etc."

During the preparations for hostilities in China, in 1859, the Director-General issued the following order: 'That a stock of quinine wine be provided, in order that a ration of it be given to the men previous to and during the unhealthy months, or when the soldiers are required to proceed up the rivers, or on being encamped in the vicinity of marshy ground. A medical officer should be present when the quinine wine is issued, and witness the same being drunk by the men.'

The committee continue their quotations at length from medical writers and travelers, showing conclusively that quinine is the great prophylactic as well as curative agent in malarious diseases. The importance of this circular at this critical period in the history of our volunteer army can scarcely be over-estimated. The great majority of the surgeons of these forces have been little accustomed to the treatment of malarious diseases. The season has arrived when the progress of the war is to transfer large bodies of troops directly into regions where malaria exists now in the most concentrated form. Unless some prophylactic is employed, these malarious fevers will decimate our susceptible army in six months, and render it impotent against an acclimated foe. Happily it is in our power to shield those who go bravely forth to meet the exigencies of war, from one of those consuming forces which threaten the Northern soldier in his progress Southward, viz: malarious diseases. We cannot sufficiently commend the Sanitary Commission for its prompt recognition of the medical necessities of our troops and the distingnished Chairman for the admirable and convincing manner in which he has presented this subject to the consideration of our authorities and the surgeons of the Army and Navy.

Pirogoff's operations at the ankle joint, has been recommended in an Order from the Medical Director of the Army of the Potomac, in preference to Chopart's, or to amputation above the ankle, in cases admitting a choice. As the operation is but little known, and the order may be extended by the Medical Directors of the Western Divisions, we re-publish, also from the *Times*, the description of the several steps as given by the author himself in an English publication:

'I commence my incision close in front of the outer malleolus, carry it vertically downwards to the sole of the foot, theu traversely across the sole, and lastly obliquely upwards to the inner malleolus, where I terminate it a couple of lines anterior to the malleolus. Thus all the soft parts are divided at once quite down to the os calcis. I now connect the outer and inner extremity of this first incision by a second semilunar incision, the convexity of which looks forward, carried a few lines anterior to the tibio-tarsal articulation. I cut through all the soft parts at once down to the bones, and then proceed to open the joint from the front, cutting through the lateral ligaments, and thus exarticulate the head of the astragalus. I now place a small narrow amputation saw obliquely upon the os calcis, behind the astragalus, exactly upon the sustentaculum tali, and saw through the os calcis, so that the saw passes into the first incision through the soft parts. Saw carefully, or the anterior surface of the tendo achillis, which is only covered by a layer of fat and a thin fibrous sheath, might be injured. I separate the short anterior flap from the two malleoli, and saw through them at the same time close to their base. I turn this flap forwards, and bring the cut surface of the os calcis in apposition with the articular surface of the tibia. If the latter be diseased it is sometimes necessary also to saw off from it a thin slice with the malleoli.'

It will be seen that Pirogoff's operation originally consisted in dividing the os calcis at right angles to its long diameter, and applying the cut surface to the articular face of the tibia, the malleoli being removed. More recently it has been slightly modified in various ways as removing the articular surface of the tibia, dividing the os calcis obliquely from above downwards and forwards, etc. It will be seen that Pirogoff's operation is, in fact, the operation for ununited bones, the two cut surfaces being placed together for the purpose of obtaining union.

Such is Pirogoff's operation. Now, as to its absolnte and relative merits, the author himself thus sums them up:

'1. The tendo achillis is not divided, and so we avoid all the disadvantages connected with its injury. 2. It also follows that the base of the posterior flap is not thinner than its apex, while the skin on the base of the flap remains ununited with the fibrous sheath of the tendo achillis. 3. The posterior flap is not cap-like, as in Syme's method, and its form is therefore less favorable to a collection of pus. 4. The leg, after my operation, appears an inch and a half (sometimes even more) longer than in the three other operations (Syme, Baudens, Roux), because the remnant of the os calcis left in the flap, as it unites with the inferior extremities of the tibia and fibula, lengthens them by an inch and a half, and 5. Serves the patient as the point of support.'

Much has been written for and against this operation. Several of the early cases were represented as terminating unfavorably by the death of the remaining portion of the os calcis, and its final separation. At one period, it was alleged that its projector had himself abandoned it. Recently, however, the statistics of the operation have been collated, and they give a more favorable impression of its value. Its mortality is fixed at about fifteen per centum, the rapidity of the cure equals that of other amputations, and the resulting limb is undeniably the best that can be obtained for direct use.

Arsenious Acid a Substitute for Quinine.—Brigade-Surgeon Turner has employed arsenious acid for twenty years in the treatment of intermittent fevers, and on account of the great drain upon the cinchona tree, its failure in India, and his strong opinion as to the equal, if not greater, value of arsenious acid in the above named diseases, he now brings the results of his experience before the profession, in a communication through Sir Ranald Martin, C. B., to the Royal Medical and Chirurgical Society, published in the *Lancet.* He considers the fears of any inconvenience or danger arising from the remedy as much exaggerated, and instances the case of a child nine months old, to whom he gave twenty minims of the arsenite of potash within ten hours, repeating the dose on the following day, with the only effect of curing an obstinate quotidian intermittent. Mr. Turner's success was so marked, that in 1860, the Director-General stated that Mr. Turner should be thanked for "drawing attention to his successful treatment of intermittent fevers by large doses of arsenic, and steps should be taken by circular, to urge an extended trial of this remedy, and reports requested." The course usually adopted by the author, was to give the arsenite of potash and compound tincture of cardamoms, of each half a drachm; gum mucilage, three drachms; camphor mixture or water, half an ounce; mix. To be given every second hour four or five times, the last to anticipate the expected paroxysm at least two hours.

Industry of John Hunter.—On a young gentleman from the country being introduced to him as one who had come to town to pursue his studies under his directions, he addressed him thus: 'Well, young gentleman, so you are come to town to be a surgeon; and how long do you intend to stay?' 'One year,' was the reply. 'Then,' said Hunter, 'I'll tell you what, that won't do; I've been here a great many years, and have worked hard, and yet I don't know the principles of the art.' After some further conversation, the student was directed to call again in an hour, to accompany the surgeon to the hospital, where, after the business of the morning was over, he said to him; 'Come to me to-morrow morning, young gentleman, and I will put you further in the way of things; come early in the morning, as soon after four as you can.' The young man kept the appointment, and at that early hour in the morning, he found Hunter at work dissecting beetles!

Here we have the secret of John Hunter's success, and of every other man's success who has attained great distinction in any art or science.

History tells us that 'his leisure hours were never allowed to remain unemployed.' He had a very different estimate of the amount to be learned, and the time and industry necessary for its accomplishment, from most people either of that or the present day.—*Dental Cosmos.*

Causes of Mild Stomatitis in Infants.—The mucous membrane of the mouth is very irritable, being accustomed only to amniotic liquor in a fœtal life, and to milk in the early stage of extra-uterine existence. Every change in the diet, therefore, the bad qualities of the maternal or artificial nipples, the use of candy, sucking bags, or alcoholic beverages, coffee, or stimulants of whatever kind, will act as irritants, producing hyperæmia or inflammation, in a more or less severe form. It is by no means common to observe very severe forms of stomatitis after all such preceding causes; on the contrary, the large majority of cases, including those depending on primary acute catarrh of the stomach, and the raising of a large quantity of gastric acid, so frequent in infantile age, are very mild.—*Prof. Jacobi, in Am. Med. Times.*

Measles Pre-disposing to Fevers.—Dr. Shipman, Surgeon to the 17th N. Y. V. M., says, in a recent letter to the *Am. Med. Times*, that "there was one thing that was to me quite a new feature. It was the effect which measles had in rendering the men susceptible to attacks of fever for a long time afterwards. While in the Park Barracks, New York, a great many of our men were affected with measles, and were sent to the hospitals. In time they recovered and joined the regiment, apparently in good health, but in the course of the summer these men were attacked without an exception, and their cases proved the most obstinate of any under treatment, and were protracted, and all assumed a typhoid character."

Effects of Climate upon Northern and Southern Troops.—Comparing the Northern soldier with the Southern, we believe the former will withstand the effects of the climate for a short campaign of a year or more better than the latter; and though the popular belief is divergent to this view, the statistics of our war with Mexico fully sustain it, and the published opinion of no less an authority than Dr. Nott, of Mobile, in the *Southern Jour. of Med. and Pharmacy*, for January, 1847, confirms it.

The statistics of the Mexican War are so remarkable, that we present them as we find them, given in a recent number of the *Evening Post:*

On April 8th, 1848, the Secretary of War made a report to the United States Senate of the losses of the volunteer forces employed in Mexico. From this, it appears that seven Northern States—Massachusetts, New York, New Jersey, Pennsylvania, Ohio, Indiana and Illinois—furnished, in the course of that war, 22,573 men. Of this force, the total loss from disease was 2,931 men; less than one-eighth of the whole. Nine Slave States—Virginia, North Carolina, South Carolina, Georgia, Alabama, Mississippi, Louisianna, Tennessee and Kentucky—furnished 22,899 men. The loss from this force from disease, and death caused from disease, was 4,315 or more than one-fifth; a very considerable difference in favor of Northern troops.—*Am. Med. Monthly.*

Russell on American Military Surgeons.—The London *Lancet* in a recent number quotes the following from one of Mr. Russell's letters to the *Times*: "The surgeons remained on the field when all others were retiring or had left. One is reported killed; six are prisoners in the hands of the enemy, engaged in attending the wounded on both sides, and an invaluable aid to the scanty medical staff of the Confederates." This is gratifying to us as a profession. It is a noble tribute to the humanity which characterizes, under whatever circumstances they may be placed, the disciples of the healing art. Far be it from us to depreciate the heroism of the combatant soldier when he rushes to meet, in mortal strife, the opposing force; but there is a heroism of a higher character—the heroism of the men who, after the fierce conflict is over, administer to the wants and necessities of those who have been wounded in the fray. Cool amidst danger, devoted to their humane efforts to preserve life, they pursue their beneficent calling amidst peril and without the hope of adequate reward. What a commentary is this upon the injustice and neglect to which medical officers attached to armies have been almost invariably subjected!

Oil of Valerian in Typhus.—During an epidemic typhus at Toulon, in 1856, characterized by stupor, somnolence, coma and great debility, the usual remedies proved of no great benefit, and M. Barrallier therefore had recourse to the essential oil of valerian. Administering it first to persons in good health, he found that it produced the following symptoms: diminution of the arterial pulsations at first, with subsequent elevation in the greater number of cases; increased heat of skin; marked perspiration with the smell of valerian; feeling of oppression in the temporal region; cephalalgia, most commonly frontal, sometimes very intense; diminution of muscular force, inaptitude for intellectual exertion; inclination to sleep; deep sleep, nausea and salivation in certain cases; dislike to food when the medicine was given in the dose of thirty or fifty centi grammes; abundant flow of urine, more highly colored than usual, with a smell of valerian. In typhus, the remedy was employed not only against the somnolence and coma occurring in the second or third week, but also at the very commencement of the disease, in order to moderate the nervous irritation. At the more advanced periods it appears to rouse the patients from their lethargic condition. Besides this, there appear as other effects: eyes widely open, intelligence more clear, correct answers to questions, increase of the arterial pulsations, with subsequent depression; diminution in the quantity of urine, and slight perspiration.—*Cincin. Lancet.*

Glycerole of Chlorate of Potash.—Two and a half drachms of the chlorate, in powder, dissolved in three ounces of glycerine, have been experimented with at Bicêtre, under the direction of Martinet, and found to possess, in that combination, remarkable disinfecting properties. Besides these, the mixture gives the pus, even when of a serous kind, a greater consistence, often like cream, and may by that action, tend to prevent the occurrence of purulent or putrid infection. The preparation is not adapted for wounds or sores of a bright red color, nor for those that are recent or of healthy appearance.—*Edinb. Med. Journ.; from Bull. Gén. de Thérap.*)

Tincture of Digitalis in Delirium Tremens.—Our exchanges continue to bear unequivocal testimony to the value of large doses of tincture of digitalis in the treatment of delirium tremens. In a late number of the *Medical Times and Gazette*, Dr. Francis E. Cavey, of Guernsey, mentions several cases of the successful use of this remedy after the entire failure of the opium treatment. He gave the tincture of digitalis in half-ounce doses, with an equal quantity of gin, and in every case found one dose sufficient. He did not find the digitalis to cause diarrhœa, vomiting, or an increased urinary secretion. One case in which the digitalis was given, was an epileptic, who had also serious disease of the heart—an aortic systolic murmur, with extensive hypertrophy. No evil effects whatever resulted in this case.

Our own experience with large doses of the tincture of digitalis in the insane department of the Philadelphia Hospital has also been very satisfactory, both in cases of delirium tremens, of which we receive a great many, and in cases of high mental excitement usual in the wards of a hospital for the insane. We usually give it in combination with an equal quantity of fluid extract of lupulin, giving a tablespoonful every two or three hours. Sleep usually supervenes after two or three doses have been administered.—*Med. and Surg. Rep.*

